# Enzymes involved in the anaerobic oxidation of n-alkanes: from methane to long-chain paraffins

**DOI:** 10.3389/fmicb.2013.00089

**Published:** 2013-05-14

**Authors:** Amy V. Callaghan

**Affiliations:** Department of Microbiology and Plant Biology, University of OklahomaNorman, OK, USA

**Keywords:** anaerobic, oxidation, alkanes, methane, paraffins

## Abstract

Anaerobic microorganisms play key roles in the biogeochemical cycling of methane and non-methane alkanes. To date, there appear to be at least three proposed mechanisms of anaerobic methane oxidation (AOM). The first pathway is mediated by consortia of archaeal anaerobic methane oxidizers and sulfate-reducing bacteria (SRB) via “reverse methanogenesis” and is catalyzed by a homolog of methyl-coenzyme M reductase. The second pathway is also mediated by anaerobic methane oxidizers and SRB, wherein the archaeal members catalyze both methane oxidation and sulfate reduction and zero-valent sulfur is a key intermediate. The third AOM mechanism is a nitrite-dependent, “intra-aerobic” pathway described for the denitrifying bacterium, ‘*Candidatus Methylomirabilis oxyfera*.’ It is hypothesized that AOM proceeds via reduction of nitrite to nitric oxide, followed by the conversion of two nitric oxide molecules to dinitrogen and molecular oxygen. The latter can be used to functionalize the methane via a particulate methane monooxygenase. With respect to non-methane alkanes, there also appear to be novel mechanisms of activation. The most well-described pathway is the addition of non-methane alkanes across the double bond of fumarate to form alkyl-substituted succinates via the putative glycyl radical enzyme, alkylsuccinate synthase (also known as methylalkylsuccinate synthase). Other proposed mechanisms include anaerobic hydroxylation via ethylbenzene dehydrogenase-like enzymes and an “intra-aerobic” denitrification pathway similar to that described for ‘*Methylomirabilis oxyfera*.’

## INTRODUCTION

Alkanes are saturated hydrocarbons that are derived from both natural and anthropogenic sources. Due to their apolar C-H σbonds, alkanes are considered to be among the least chemically reactive organic compounds. The activation or functionalization of alkanes is initiated via cleavage of a C-H bond. Aerobic microorganisms achieve this step via monooxygenase or dioxygenase enzymes, in which oxygen serves as both the physiological terminal electron acceptor and as a reactant (for review of mechanisms and enzymes see Austin and Groves, 2011). The role of oxygen in the functionalization of alkanes led to the belief for many years that anaerobic microorganisms would be unable to activate and utilize these compounds as growth substrates. However, research during the last 25 years has demonstrated that anaerobic microorganisms have their own novel mechanisms of activating alkanes.

## ANAEROBIC OXIDATION OF METHANE

The shortest alkane, methane, is the most environmentally relevant hydrocarbon due to its biological production by methanogenic archaea in freshwater systems, swamps, landfills, marine sediments and seeps, rice fields, and other anaerobic environments, as well as its role as a greenhouse gas (Conrad, 2009). Anaerobic methane oxidation (AOM) is thought to account for the removal of over 300 Tg of methane per year in oceanic systems (Hinrichs and Boetius, 2002; Reeburgh, 2007). Therefore, from the perspective of climate change, AOM serves as a significant greenhouse “sink.” AOM can be coupled to the reduction of sulfate, nitrate and nitrite (for reviews see Knittel and Boetius, 2011; Shen et al., 2012), manganese (Beal et al., 2009), and iron (Beal et al., 2009; Crowe et al., 2011; Sivan et al., 2011; Amos et al., 2012; Zhu et al., 2012). Although the mechanisms of aerobic methane oxidation are well-described (Austin and Groves, 2011), the mechanisms of AOM have been hotly debated.

### REVERSE METHANOGENESIS

**FIGURE 1:**
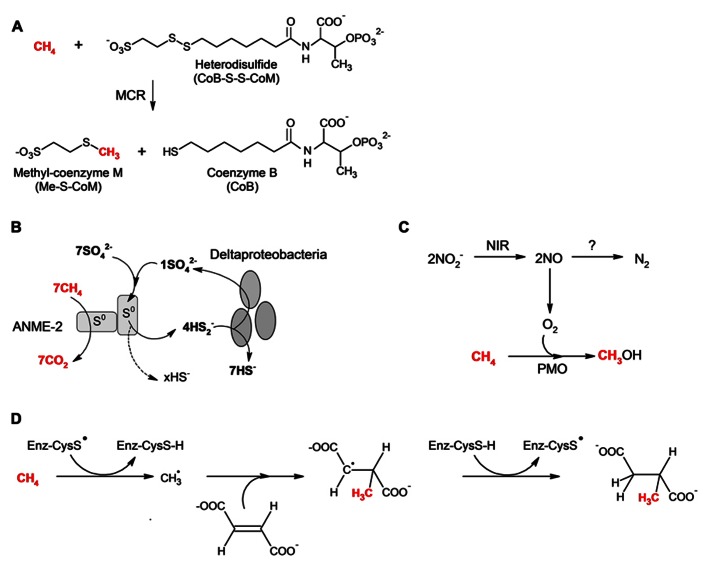
**Proposed pathways for the anaerobic oxidation of methane (AOM) via:(A) Reverse methanogenesis (adapted from Scheller et al., 2010a,b); (B) Zero-valent sulfur as a key intermediate in AOM (adapted from Milucka et al., 2012); (C)Nitrite-dependent anaerobic methane oxidation (“intra-aerobic denitrification” adapted from (Ettwig et al., 2010); and (D) Methane addition to fumarate (adapted from Beasley and Nanny, 2012)**. Enzyme nomenclature: methyl-coenzyme M reductase (MCR); nitrite reductase (NIR); particulate methane monooxygenase (PMO); and the putative thiyl radical of the alkylsuccinate/methylalkylsuccinate synthase-like enzyme (Enz-CysS).

To date, several studies have revealed that AOM coupled to sulfate reduction is mediated by consortia of archaeal anaerobic methane oxidizers (ANME-1, ANME-2, and ANME-3) and sulfate-reducing bacteria (SRB) (Knittel and Boetius, 2011). AOM with sulfate as the terminal electron acceptor appears to proceed via “reverse methanogenesis” (Zehnder and Brock, 1979; Hoehler et al., 1994; Hallam et al., 2004) and is catalyzed by a homolog of methyl-coenzyme M reductase (MCR), the Ni-containing enzyme responsible for the last step of methanogenesis (Krüger et al., 2003; Scheller et al., 2010a; **Figure 1A**). MCR homologues have been found in high concentrations in methanotrophic archaea associated with SRB (Hallam et al., 2003; Krüger et al., 2003; Heller et al., 2008; Mayr et al., 2008; Nunoura et al., 2008). In a recent study, an ANME-1 MCR complex was isolated from a Black Sea microbial mat, and the X-ray structure was reported (Shima et al., 2012). Compared to the methanogenic MCR, the ANME-1 MCR complex is similar in overall structure and contains coenzyme M and coenzyme B. However, it differs from the methanogenic MCR in that it contains an F_430_ variant, as well as a cysteine-rich region and an altered post-translational amino acid modification pattern (Shima et al., 2012). Although not clearly elucidated, the role of the cysteine-rich region between the F_430_ and the protein surface may be to operate as a redox-relay system for electron or H^+^/e^-^ transport for the reduction of ANME-1 MCR from the inactive Ni^2^^+^ to the active Ni^1^^+^ oxidation state (Mayr et al., 2008; Shima et al., 2012), whereas, the altered post-translational amino acid modifications may reflect phylogenetic adaptation to environmental conditions (Shima et al., 2012) based on the distribution of different ANME groups within microbial mats (Krüger et al., 2008).

The “reversibility” of MCR is supported by thermodynamic and kinetic considerations (Thauer, 2011), as well as activity assays with purified MCR from *Methanothermobacter marburgensis* (Scheller et al., 2010a). However, there are still several hypotheses regarding the reaction intermediates of both methane formation and methane oxidation by MCR. The two main competing theories argue either for an organometallic methyl-Ni^*III*^F_430_ intermediate (Ermler et al., 1997; Dey et al., 2010) or the formation of a methyl radical and a CoM-S-Ni^*II*^F_430_ intermediate (Pelmenschikov et al., 2002; Pelmenschikov and Siegbahn, 2003). The latter was challenged in an experimental study which predicted the formation of (σ-alkane)-NiF_430_ and (H)(alkyl)NiF_430_ complexes as intermediates (Scheller et al., 2010b). However, recent active site models of the MCR X-ray crystal structure (PDB code 1HBN) from *Methanothermobacter thermautotrophicus* support the theory that methane oxidation proceeds via a methyl radical and a CoM-S-Ni^*II*^F_430_ intermediate (Chen et al., 2012).

### ALTERNATIVE AOM MECHANISMS UNDER SULFATE-REDUCING CONDITIONS

There has been tremendous debate regarding the interspecies intermediates of sulfate-dependent AOM, and several mechanisms have been proposed for consortia of ANME and SRB. One hypothesis proposes the formation of acetic acid and H_2_ from methane and water by methane-oxidizing archaea, with subsequent utilization of the acetic acid and H_2_ by SRB (Valentine and Reeburgh, 2000). Another variation of this process was previously proposed as “reverse acetoclastic methanogenesis” in which methane-oxidizing archaea produce acetate from CO_2_ and CH_4_, and the acetate is utilized by SRB (Zehnder and Brock, 1980; Hoehler et al., 1994). More recently, a “methylogenesis” mechanism was proposed, in which methanethiol (MeSH) serves as an interspecies compound that transfers methane-derived carbon from the methanotroph to the SRB (Moran et al., 2008). With respect to the latter, there is some evidence that methanethiol inhibits AOM (Moran et al., 2008; Meulepas et al., 2010) and sulfate reduction (Meulepas et al., 2010). Therefore, the role of methylsulfides is still unclear. Additionally, several studies have provided experimental evidence and theoretical predictions that compounds such as acetate, hydrogen, formate, methanol, and carbon monoxide are unlikely to serve as interspecies compounds based on their effects on sulfate reduction and AOM, their diffusion distances, and thermodynamic considerations (see Meulepas et al., 2010 and references therein). Finally, extracellular electron transfer between archaea and deltaproteobacteria via nanowires (Reguera et al., 2005; Gorby et al., 2006) or direct electron transfer via multi-heme *c*-type cytochromes (Meyerdierks et al., 2010) has also been proposed. These processes, however, would require the archaea and deltaproteobacteria to be in direct physical contact with each other.

### NEW MODEL FOR AOM MECHANISM UNDER SULFATE-REDUCING CONDITIONS: ZERO-VALENT SULFUR AS A KEY INTERMEDIATE

The studies focusing on the identification of AOM intermediates under sulfate-reducing conditions (see above) have mainly been predicated on the hypothesis that the ANME/SRB consortia catalyze AOM via an *obligate* syntrophic mechanism (for review see Knittel and Boetius, 2009). However, a new study of an enrichment culture obtained from sediments of the Mediterranean mud volcano Isis challenges that paradigm and also provides new insight to potential intermediates that may be transferred from the archaeal members to the SRB (Milucka et al., 2012). The culture consists of ANME-2 and bacteria belonging to the *Desulfosarcina*/*Desulfococcus* (DSS) cluster (Schreiber et al., 2010), and evidence suggests that the ANME-2 assimilate dissolved inorganic carbon (DIC) and catalyze both the anaerobic oxidation of methane and the reduction of sulfate to zero-valent sulfur (S^0^) and possibly to sulfide. Although experimental evidence is not yet available, the reduction of sulfate by ANME-2 is proposed to proceed via a non-canonical enzymatic pathway in which the electron donor may be the formylmethanofuran (MFR)/CO_2_ + MFR redox couple. The zero-valent sulfur can react with sulfide to form polysulfides, such as disulfide. The DSS then couple disproportionation of disulfide to sulfide and sulfate (**Figure 1B**) with autotrophic carbon assimilation. Presumably, the disproportionation reaction in DSS could be catalyzed by enzymes such as sulfate adenylyltransferase (SAT) and adenylylsulfate reductase (APR), which have been shown to be involved in the disproportionation of compounds such as elemental sulfur, thiosulfate, and sulfite (Krämer and Cypionka, 1989). The sulfate generated by the DSS can be partly reused by the ANME-2. Stoichiometrically, no net sulfate production occurs. Given these findings, it appears as though the ANME-2 are not necessarily dependent upon the sulfate-reducing partner and may be able to associate with any bacteria capable of scavenging disulfide and coupling it to energy generation and growth. This has important implications with respect to the biogeochemistry of methane oxidation and sulfur cycling as well as studies investigating interspecies compounds that are indicative of an obligate syntrophic AOM mechanism (see above).

### NITRITE-DEPENDENT ANAEROBIC METHANE OXIDATION (“INTRA-AEROBIC DENITRIFICATION”)

Anaerobic methane oxidation coupled to denitrification through “reverse methanogenesis” was first proposed for a consortium (“Twente”) enriched from Twentekanaal sediment (Netherlands) (Raghoebarsing et al., 2006). The culture consisted of a bacterium belonging to the candidate division, “NC10,” and archaea distantly related to *Methanosaeta* and ANME–II (Raghoebarsing et al., 2006; Ettwig et al., 2009). Subsequent investigations of the “Twente” culture showed AOM in the *absence* of the archaea (Ettwig et al., 2008, 2009), and further characterization of “Twente” and another culture (“Ooij”)(Ettwig et al., 2009) revealed microbial populations dominated by the denitrifying bacterium, ‘*Candidatus Methylomirabilis oxyfera*’ (Ettwig et al., 2010). Genome assembly obtained from metagenomic analyses shows that ‘*Methylomirabilis oxyfera*’ contains genes encoding enzymes for the complete pathway for aerobic methane oxidation, including a particulate monooxygenase (pMMO). The genome also contains several genes for denitrification, but lacks the genes encoding the enzymes for the reduction of nitrous oxide to dinitrogen (*nosZDFY*), an interesting finding given the observed production of N_2_ in the “Twente” and “Ooij” cultures during AOM (Raghoebarsing et al., 2006; Ettwig et al., 2008, 2009). ‘*Methylomirabilis oxyfera*’ also lacks genes encoding homologs of MCR or alkane-activating glycyl radical enzymes (Ettwig et al., 2010). Isotopic labeling experiments suggest that AOM by ‘*Methylomirabilis oxyfera*’ proceeds via reduction of nitrite to nitric oxide, followed by the conversion of two nitric oxide molecules to dinitrogen and molecular oxygen. The latter can be used to functionalize the methane via the pMMO (**Figure 1C**). Based on stoichiometry (3CH_4_ + 8NO_2_^-^ + 8H^+^ → 3CO_2_ + 4N_2_ + 10H_2_O), four molecules of O_2_ could be generated from eight molecules of nitrite, of which only three are required for methane activation by pMMO (Wu et al., 2011). Based on oxygen uptake and inhibition experiments with cell extracts and UV–visible absorption spectral characteristics and electron spin resonance (EPR) spectroscopy of solubilized membranes, the remaining oxygen might be consumed by a membrane-bound *bo*-type terminal oxidase (Wu et al., 2011).

### METHANE ADDITION TO FUMARATE

The addition of non-methane alkanes to fumarate (i.e., fumarate addition) is a prevalent anaerobic alkane activation mechanism observed under nitrate- and sulfate-reducing conditions (see below). Although the majority of studies have focused on longer-chain alkanes, a few investigations have revealed that short alkanes, such as propane, are also added to fumarate terminally or subterminally under sulfate-reducing conditions (Kniemeyer et al., 2007; Savage et al., 2010). These findings are supported by field metabolomic studies of petroleum reservoirs and coalbeds in which low molecular weight gases such as methane are prevalent and low molecular weight alkylsuccinates, including methylsuccinate, have been detected (Duncan et al., 2009; Gieg et al., 2010; Wawrik et al., 2012). Due to the complex mixture of hydrocarbons in oil reservoirs and coalbeds, it is possible that the low molecular weight alkylsuccinates are derived from cometabolic hydrocarbon metabolism, as has been observed for toluene in anaerobic alkane-degrading bacteria (Rabus et al., 2011). However, the detection of methylsuccinate is intriguing nonetheless because it suggests that AOM via fumarate addition may be possible (**Figure 1D**). Thermodynamically, however, there are several considerations with respect to the formation of the methyl radical and the terminal electron accepting conditions. The formation of a methyl radical (439 kJ mol^-^^1^) (Lide, 2003) would be at the expense of the glycyl radical formation (350 kJ mol^-^^1^) (Armstrong et al., 1996). The difference in dissociation energies (90 kJ) is considerably larger than that for other alkane substrates (~60 kJ) (Thauer and Shima, 2008), and at first glance, it would not appear that the transition state of the enzyme could overcome such a barrier. A study using quantum chemical calculations to investigate the energetics of methane addition to fumarate (Beasley and Nanny, 2012) also predicts that the initial reaction (i.e., homolytic cleavage of the C-H bond in methane by the methylethyl radical) is unfavorable, but that all other steps in the proposed reaction are favorable, with an overall energy change that is exothermic. Given that glycyl radical enzymes are functional dimers, it is possible that the initial energy barrier might be overcome via coupling the endergonic steps of the catalytic cycle in one active site with exergonic steps in the catalytic cycle of the second active site (Thauer and Shima, 2008).

Whether AOM via fumarate addition proceeds with sulfate versus nitrate or nitrite as the terminal electron acceptor has also been debated. It is hypothesized that the majority or all of the free energy generated from this process with sulfate as the terminal electron acceptor (δG^°^^′^ = -21 kJ/mol) would be dissipated as heat in the initial activation step, which would not allow enough energy for subsequent reactions, whereas the free energy change using nitrate or nitrite as a terminal electron acceptor is more energetically favorable (Thauer and Shima, 2008).

## ANAEROBIC OXIDATION OF NON-METHANE ALKANES

### ALKANE ADDITION TO FUMARATE

**FIGURE 2:**
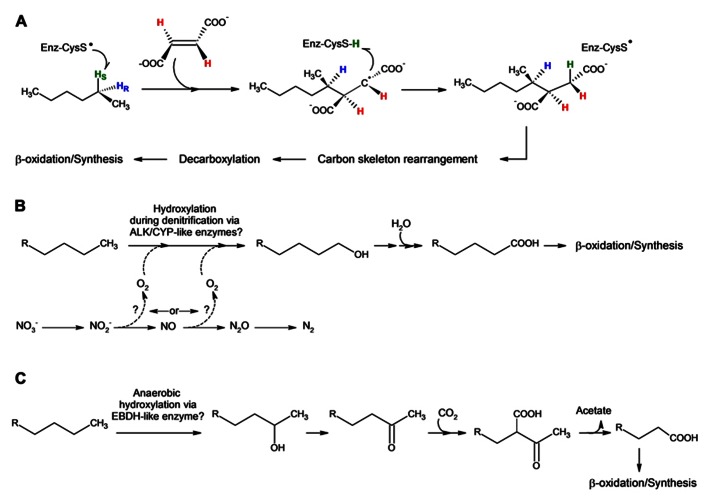
**(A)** Proposed pathway and stereochemistry for the anaerobic activation and degradation of hexane via subterminal addition to fumarate (adapted from Jarling et al., 2012); **(B)** Proposed pathway of activation and degradation of non-methane alkanes via “intra-aerobic denitrification” (adapted from Zedelius et al., 2011); and **(C)** Proposed pathway of activation and degradation of non-methane alkanes via anaerobic hydroxylation by *Desulfococcus oleovorans* Hxd3 (Sünwoldt et al., 2012). Enzyme nomenclature: putative thiyl radical of the alkylsuccinate/methylalkylsuccinate synthase-like enzyme (Enz-CysS); alkane monooxygenase (ALK); cytochromes P450 (CYP); and ethylbenzene dehydrogenase (EBDH).

One of the most well characterized mechanisms of anaerobic alkane activation and degradation is the addition of non-methane alkanes across the double bond of fumarate to form alkyl-substituted succinates. This was first demonstrated in a sulfate-reducing, dodecane-utilizing enrichment culture (Kropp et al., 2000) and was subsequently found to be applicable to a range of *n*-alkane and cycloalkane substrates (C_3_–C_1__6_) in several sulfate-reducing and nitrate-reducing isolates and cultures (Rabus et al., 2001; Wilkes et al., 2002, 2003; Rios-Hernandez et al., 2003; Cravo-Laureau et al., 2005; Davidova et al., 2005; Callaghan et al., 2006; Kniemeyer et al., 2007; Musat et al., 2010). Although subterminal addition of *n*-alkanes to fumarate appears to be a prevalent feature of the fumarate addition mechanism (**Figure 2A**), other modes include C3 addition (Rabus et al., 2001) and C1 addition (Kniemeyer et al., 2007), both of which have been proposed as side-reactions. The alkyl-substituted succinates are further degraded via carbon-skeleton rearrangement followed by decarboxylation and β-oxidation (Wilkes et al., 2002).

Analogous to the glycyl radical mechanism of benzylsuccinate synthase (BSS) and its homologs, which catalyze the addition of aromatic hydrocarbons to fumarate (for reviews see Boll and Heider, 2010; Meckenstock and Mouttaki, 2011), alkane activation is presumably catalyzed by the glycyl radical enzyme, alkylsuccinate synthase (ASS) (Callaghan et al., 2008b) or methylalkylsuccinate synthase (MAS) (Grundmann et al., 2008), for which the sulfate-reducing strain *Desulfatibacillum alkenivorans* AK-01 and the denitrifying strain ‘*Aromatoleum*’ HxN1 currently serve as model strains, respectively. Comparison of *ass/mas* genes to *bss* genes has revealed several similarities. Both AK-01 and HxN1 contain genes encoding putative glycyl radical activating enzymes, belonging to the radical *S*-adenosylmethionine (SAM) superfamily. Similar to BSS activase, which generates a glycyl radical on the catalytic subunit of BSS (Leuthner et al., 1998; Krieger et al., 2001; Verfürth et al., 2004), it is thought that the ASS activase is responsible for the generation of the glycyl radical on the catalytic subunit of the alkane-activating enzyme. Subsequent generation of a thiyl radical on a conserved cysteine residue (Selmer et al., 2005) would result in the abstraction of an H atom from the hydrocarbon substrate (Heider et al., 1998). In the case of BSS, the reaction results in the stereospecific formation of *R*(+)-benzylsuccinate (Beller and Spormann, 1998; Leutwein and Heider, 1999), wherein the initially abstracted H atom is transferred to the same face of fumarate (Qiao and Marsh, 2005). Unlike benzylsuccinate, however, methylalkylsuccinic acids contain two chiral carbons. Metabolomic investigations of numerous sulfate- and nitrate-reducing cultures utilizing *n*-alkanes show the formation of two diastereomers of the methylalkylsuccinic acid metabolites (Kropp et al., 2000; Rabus et al., 2001; Wilkes et al., 2002; Cravo-Laureau et al., 2005; Callaghan et al., 2006), and gas chromatography-mass spectrometry (GC-MS) analysis of a sulfate-reducing enrichment culture utilizing ethylcyclopentane resolved up to five peaks corresponding to the requisite ethylcyclopentylsuccinic acids (Rios-Hernandez et al., 2003). Recent stereochemical investigations of ‘*Aromatoleum*’ HxN1growing on *n*-hexane showed only the formation of (2R, 1^′^R) and (2S, 1^′^R) isomers of 1-methylpentylsucccinate and that the initial step in activation is via abstraction of the pro-*S* hydrogen from C_2_ of hexane, with subsequent addition of the alkyl species intermediate to fumarate on the opposite face from which the hydrogen atom was abstracted (**Figure 2A**; Jarling et al., 2012). It is also proposed that the initial 1-methylpentylsuccinate isomer is epimerized to generate a diastereomer that then undergoes the reported carbon-skeleton rearrangement and subsequent decarboxylation (Jarling et al., 2012).

The discoveries of ASS and MAS have enabled the interrogation of hydrocarbon-impacted environments as well as isolates and enrichment cultures (Callaghan et al., 2010; Kolukirik et al., 2011; Andrade et al., 2012; Li et al., 2012; Mbadinga et al., 2012; Wang et al., 2012; Wawrik et al., 2012; Zhou et al., 2012; Cheng et al., 2013). In addition to alkanes of medium chain lengths, ASS/MAS enzymes appear to play a role in the degradation of very short alkanes (eg., propane) (Kniemeyer et al., 2007; Callaghan et al., 2010; Savage et al., 2010), and recent investigations of anaerobic biodegradation of solid paraffins under methanogenic conditions suggest that microorganisms may also activate paraffin waxes via fumarate addition (Callaghan et al., 2010). With respect to the latter, the detection and expression of several *assA* genotypes was demonstrated in a methanogenic consortium utilizing octacosane (C_28_) (Davidova et al., 2011). However, the requisite metabolites have not been identified in this study or in other studies examining alkane activation under methanogenic conditions for which *assA* genotypes have been detected (Mbadinga et al., 2012; Wang et al., 2012; Zhou et al., 2012; Aitken et al., 2013; Cheng et al., 2013). It has been hypothesized that either the metabolites that result from fumarate addition do not accumulate to detectable levels under the slow-growing conditions and/or that other mechanisms, such as hydroxylation, may be important (Aitken et al., 2013).

### INTRA-HYDROXYLATION

An “intra-aerobic” pathway of alkane oxidation similar to that of ‘*Methylomirabilis oxyfera*’ has also been proposed to be involved in the functionalization of *n*-hexadecane by the Gammaproteobacterium HdN1 (**Figure 2B**; Zedelius et al., 2011). Unlike ‘*Methylomirabilis oxyfera*,’ HdN1 contains genes for the complete denitrification pathway. The genome of strain HdN1 does not contain genes that encode an alkane-activating glycyl radical enzyme, but does contain genes that may encode a di-iron monooxygenase, a P_450_-type monooxygenase and a putative third type of monooxygenase (Zedelius et al., 2011). Consistent with the absence of an alkane-activating glycyl radical enzyme, alkyl-substituted succinates were not detected via metabolite profiling, but 1-hexadecanol was detected when the anaerobic culture was exposed to air. Based on these findings and growth experiments, the authors proposed a mechanism in which NO_2_^-^, NO or an unknown product of NO_2_^-^ reduction may be required for alkane activation. Specifically, the dismutation of NO_2_^-^ [ΔG^°^^′^= -55.2 kJ (mole O_2_)^-^^1^] or NO [ΔG^°^^′^= -173.1 kJ (mole O_2_)^-^^1^)] would provide O_2_, which could then be used to hydroxylate the alkane via one of the putative monooxygenases. Alternatively, N-O species could behave as strongly oxidizing electron acceptors to generate a reactive state of a factor or enzyme site that is involved in the activation of the alkane, or an N-O species may be directly involved in the activation of the alkane (Zedelius et al., 2011).

### ANAEROBIC HYDROXYLATION FOLLOWED BY CARBOXYLATION

Finally, there appears to be at least one additional pathway of anaerobic alkane oxidation. Early studies of the sulfate-reducing bacterium *Desulfococcus oleovorans* Hxd3 demonstrated that incubation with an alkane with an odd number of C atoms yielded predominantly fatty acids with an even number of C atoms, and vice versa (Aeckersberg et al., 1998; So et al., 2003). This is in contrast with what is observed in microorganisms that activate alkanes via fumarate addition (So and Young, 1999). Stable isotope studies of strain Hxd3 incubated with NaH^13^CO_3_ and [1,2-^13^C_2_]hexadecane were indicative of incorporation of carbon derived from bicarbonate at C3 and elimination of the C1 and C2 carbon atoms (So et al., 2003). Subsequently, evidence of this pathway was also observed in nitrate- and sulfate-reducing enrichment cultures utilizing *n*-hexadecane (Callaghan et al., 2006, 2009). However, although it was hypothesized that carboxylation at C3 may be the first step in degradation, the corresponding carboxylated intermediate, 2-ethylpentadecanoic acid, was not detected in any of the above studies. Under standard conditions, the direct carboxylation of alkanes is an endergonic process (ΔG^°^^′^= +28 kJ/mol) (Thauer and Shima, 2008). Thus, carboxylation as the first step in alkane degradation in Hxd3 has been debated for almost 10 years.

With the continual evolution of sequencing technologies, however, genome-enabled analyses are yielding new hypotheses. Consistent with the above experimental observations, the genome of Hxd3 does not contain genes encoding an alkane-activating glycyl radical enzyme. Interestingly, the genome contains genes that encode an ethylbenzene dehydrogenase-like complex (Callaghan et al., 2008a), similar to that found in denitrifying strains *Aromatoleum aromaticum* EbN1 (Kniemeyer and Heider, 2001) and *Azoarcus* sp. strain EB1 (Johnson and Spormann, 1999; Johnson et al., 2001). Ethylbenzene dehydrogenase is a molybdenum-cofactor-containing enzyme of the dimethylsulfoxide reductase family that catalyzes the anaerobic hydroxylation of ethylbenzene (Ball et al., 1996; Rabus and Heider, 1998). Based on these findings, it is possible that the activation of alkanes by Hxd3 may also occur via anaerobic hydroxylation. Analogous to the steps involved in the anaerobic hydroxylation of ethylbenzene, hydroxylation of the alkane at C2 would produce a secondary alcohol that could be further oxidized to a ketone, similar to the formation of acetophenone during ethylbenzene degradation (**Figure 2C**; Ball et al., 1996; Rabus and Heider, 1998) Subsequent transformation of the ketone may involve carboxylation at C3, with elimination of the C1 and C2 carbons, to produce a fatty acid that is one carbon shorter than the parent alkane. The latter would be consistent with previous studies (So and Young, 1999; Callaghan et al., 2006, 2009). Proteomic investigations of alkane-degrading cells of Hxd3 implicate the putative ethylbenzene dehydrogenase (Sünwoldt et al., 2012). However, further study is needed to elucidate the transformation reactions that would ultimately result in the formation of the observed fatty acids.

## CONCLUSION

The anaerobic oxidation of alkanes plays an important role in the biogeochemical cycling of methane and the bioremediation of hydrocarbon-impacted environments. As we look to the future, advances in next-generation sequencing and annotation will facilitate genome-enabled transcriptomic and proteomic investigations of anaerobic alkane oxidation. The complete genome sequences of several model alkane utilizers are now publicly available and include: *Desulfatibacillum alkenivorans* AK-01, *Desulfococcus oleovorans* Hxd3, ‘*Candidatus Methylomirabilis. oxyfera*,’ and the Gammaproteobacterium, strain HdN1. Future work will rely on these model organisms for the purification and characterization of relevant enzymes.

## Conflict of Interest Statement

The author declares that the research was conducted in the absence of any commercial or financial relationships that could be construed as a potential conflict of interest.
